# Structural Basis for Linezolid Binding Site Rearrangement in the *Staphylococcus aureus* Ribosome

**DOI:** 10.1128/mBio.00395-17

**Published:** 2017-05-09

**Authors:** Matthew J. Belousoff, Zohar Eyal, Mazdak Radjainia, Tofayel Ahmed, Rebecca S. Bamert, Donna Matzov, Anat Bashan, Ella Zimmerman, Satabdi Mishra, David Cameron, Hans Elmlund, Anton Y. Peleg, Shashi Bhushan, Trevor Lithgow, Ada Yonath

**Affiliations:** a Infection & Immunity Program, Biomedicine Discovery Institute & Department of Microbiology, Monash University, Clayton, Australia; b Department of Structural Biology, Weizmann Institute of Science, Rehovot, Israel; c The Clive and Vera Ramaciotti Centre for Structural Cryo-Electron Microscopy, Department of Biochemistry and Molecular Biology, Monash University, Victoria, Melbourne, Australia; d School of Biological Sciences, Nanyang Technological University, Singapore, Singapore; e Infection & Immunity Program, Biomedicine Discovery Institute & Department of Biochemistry and Molecular Biology, Monash University, Clayton, Australia; f Department of Infectious Diseases, Alfred Hospital, Prahran, Australia; g NTU Institute of Structural Biology, Nanyang Technological University, Singapore, Singapore; National Jewish Hospital; Louis Stokes Veterans Affairs Medical Center

**Keywords:** staphylococcus, antibiotic resistance, ribosomal mutations

## Abstract

An unorthodox, surprising mechanism of resistance to the antibiotic linezolid was revealed by cryo-electron microscopy (cryo-EM) in the 70S ribosomes from a clinical isolate of *Staphylococcus aureus*. This high-resolution structural information demonstrated that a single amino acid deletion in ribosomal protein uL3 confers linezolid resistance despite being located 24 Å away from the linezolid binding pocket in the peptidyl-transferase center. The mutation induces a cascade of allosteric structural rearrangements of the rRNA that ultimately results in the alteration of the antibiotic binding site.

## IMPORTANCE

The growing burden on human health caused by various antibiotic resistance mutations now includes prevalent *Staphylococcus aureus* resistance to last-line antimicrobial drugs such as linezolid and daptomycin. Structure-informed drug modification represents a frontier with respect to designing advanced clinical therapies, but success in this strategy requires rapid, facile means to shed light on the structural basis for drug resistance (D. Brown, Nat Rev Drug Discov 14:821–832, 2015, https://doi.org/10.1038/nrd4675). Here, detailed structural information demonstrates that a common mechanism is at play in linezolid resistance and provides a step toward the redesign of oxazolidinone antibiotics, a strategy that could thwart known mechanisms of linezolid resistance.

## OBSERVATION

**FIG 1:**
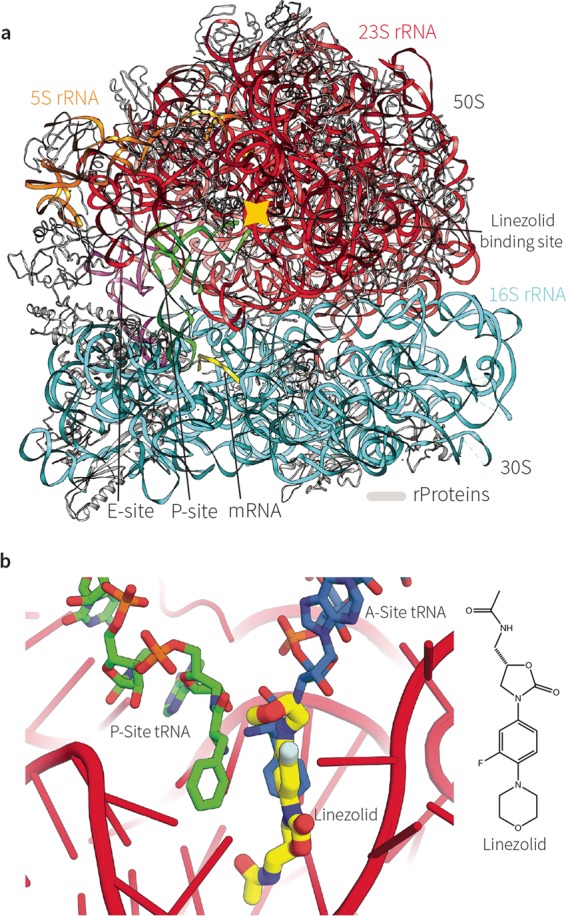
Linezolid binding and protein synthesis in *S. aureus*. (a) The overall structure of the 70S ribosome from *S. aureus* (Lin^S^). (b) (Left panel) The 23S rRNA of the catalytic site is shown in red, the amino-acylated end of the A-site tRNA is shown in green, and the P-site tRNA is shown in orange. The antibiotic linezolid, overlapping the position that the A-site tRNA would otherwise occupy, is shown in yellow. Upon linezolid binding, no amino-acylated tRNAs can enter the active site and form new peptide bonds. The cartoon represents a composite of two PDB entries: 4WFA (24) and 4V5D (38). (Right panel) The chemical structure of linezolid.

Ribosomes (Fig. 1a) are the cellular nanomachines responsible for protein synthesis (1, 2). As such, the bacterial ribosome is targeted by over 40% of the antibiotics (3) in clinical use. While the acquisition of resistance to many of these antibiotics is of great current concern, two strategies stand out for clinically relevant “push back” at drug resistance phenotypes: (i) the design of novel antibiotics and (ii) the use of structure-informed drug engineering to modify current antibiotics to produce variant antibiotics by pinpointing and then overcoming steric factors in drug-resistant ribosomes (4). Linezolid—the first fully synthetic antibiotic—was introduced as a new drug in the clinic in 2000 (5, 6). It inhibits bacterial protein synthesis by binding in the peptidyl transferase center (PTC), the active site of the large 50S ribosomal subunit (Fig. 1b). Linezolid binding leads to steric hinderance that selectively modulates tRNA binding into the A-site of the ribosome (7, 8), and crystal structures reveal that the bound linezolid adopts similar orientations in the A-sites of ribosomes from bacterial species as diverse as *Deinococcus radiodurans*, *Haloarcula marismortui*, *Staphylococcus aureus*, and *Escherichia coli* (9).

The acquisition of linezolid resistance by methicillin-resistant *Staphylococcus aureus* (MRSA) is now of global concern (10–12). In the absence of new antibiotics, reengineering of a drug like linezolid is potentially an attractive strategy (4). In terms of genome-based characterization, resistance to linezolid is conferred by numerous rRNA mutations, with particular prevalence seen in the mutations C^2576^U, U^2500^A, C^2190^U, and G^2603^U (*E. coli* 23S rRNA numbering is used throughout) (10, 13–16) and in the acquisition by lateral transfer of the *cfr* gene encoding a methyltransferase that modifies A^2503^ in the 23S rRNA. Strains of MRSA with the combination of G^2603^U and the *cfr* gene are now of particular concern in Asia (10). Seemingly at odds with the observation that a discrete set of rRNA mutations are causative for linezolid resistance, a recent increase in the levels of isolates of linezolid-resistant pathogens, including MRSA, with mutations in ribosomal protein uL3, a ribosomal protein positioned at a location distant from that of the PTC, has been reported (15, 17). To rationalize these observations, current biochemically based hypothetical models suggest various sterically different solutions to resist linezolid binding (15, 18). A scenario such as this, with various structurally distinct mechanisms at play, would make unfeasible any attempt to reengineer a form of linezolid that could reliably act against linezolid-resistant pathogens.

**FIG 2:**
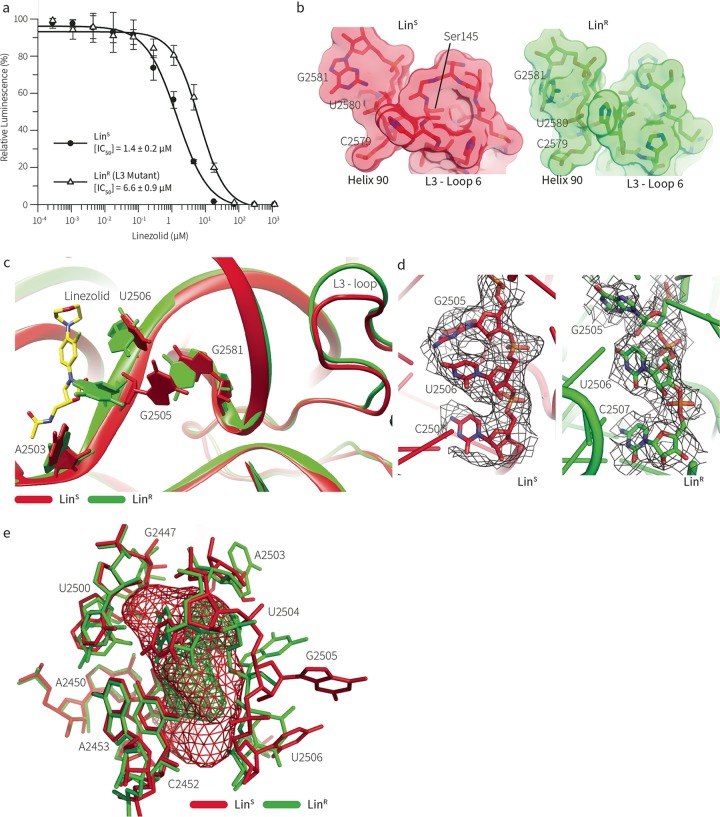
A structural clash that prevents linezolid binding in Lin^r^ MRSA. (a) *In vitro* (IC_50_) cell-free transcription-translation assay. Data are plotted as the amount of protein synthesis (measured by luciferase translation) versus the concentration of linezolid (in micrograms per milliliter). (b) Representation of the region of uL3 around the site of the ΔSer145 mutation, viewed in the same orientation. The Lin^s^ structure is shown on the left (red); the Lin^r^ structure is shown on the right (green). The portion of uL3 that interacts with rRNA in “helix 90” is shown. The Lin^r^ structure reveals a contraction in the loop of uL3, visible here by the repositioning of His146 (uL3) to become His145 (uL3) in the LinR ribosome, altering the interaction of this loop with helix 90 of the 23S rRNA. (c) The cryo-EM structure of the 70S ribosome from Lin^s^, showing the linezolid position (yellow) and its interaction with 23S rRNA nucleotide G^2505^ (the position of linezolid is from PDB 4WFA). The cryo-EM structure of the 70S ribosome from Lin^r^ is overlaid in green. Deletion of a single amino acid in the uL3 rProtein of the Lin^r^ ribosome changes the part of the protein that interacts with an rRNA helix, a helix which in turn makes a direct contact with linezolid. This structural change modifies the position of G^2505^ in the drug binding pocket, contracting this region and providing fewer contacts to stabilize drug binding. (d) Electron density (drawn at 3σ) around the rRNA nucleotides closest to the linezolid binding site in both the Lin^s^ (red, left panel) and Lin^r^ (green, right panel), showing the change in orientation of these nucleotides due to the mutation in uL3. (e) Overlay of the cryo-EM structures of the Lin^s^ and Lin^r^ 70S ribosomes around the binding cavity of the linezolid antibiotic. The cavity available for linezolid binding is shown in the colored mesh. The remodeled Lin^r^ binding site (green) is more constricted and less permissive of linezolid binding.

We examined a linezolid-resistant (Lin^r^) clinical isolate of MRSA from an Australian hospital by single-particle cryo-electron microscopy (cryo-EM). The antibiotic susceptibility of the strain was assessed by *in vitro* transcription-translation assays using bacterial cell extracts. These data quantify susceptibility to inhibition of translation, with ribosomes from the Lin^r^ strain showing a more than 4-fold increase in the 50% inhibitory concentration (IC_50_) compared to ribosomes from the type (Lin^s^) strain, ATCC 35556 (Fig. 2a). Genome sequencing of the Lin^r^ strain revealed that linezolid resistance was due not to the presence of the *cfr* gene or to any mutation in the rRNA genes but to the presence of a single amino acid deletion (Δ^S145^) in ribosomal protein (rProtein) uL3 (see Fig. S1 in the supplemental material). The mutation in uL3 sits at a location greatly distal to the PTC (>20 Å away), and we sought to use high-resolution structural data to reconcile the architectural constraints in the ribosome with the drug resistance phenotype.

10.1128/mBio.00395-17.3 FIG S1  The Lin^r^ mutation in *rpsC*. Download FIG S1, PDF file, 0.4 MB. Copyright © 2017 Belousoff et al. 2017 Belousoff et al. This content is distributed under the terms of the Creative Commons Attribution 4.0 International license.

Using a rapid workflow for cryo-EM data acquisition and data processing, single-particle reconstructions enabled determination of 70S ribosome structures from the Lin^s^ and Lin^r^ strains of *S. aureus* (Fig. S2, Fig. S3, Table S1). Ribosomes were extracted from cultures at mid-log phase and purified from bacterial cell extracts by ultracentrifugation coupled with hydrophobic interaction chromatography and sucrose gradient fractionation (see Text S1 in the supplemental material). At the time of this study, no high-resolution structural data were available for 70S ribosomes from *S. aureus*. Processing of the raw cryo-EM data was performed with RELION software (19), resulting in a 3.9-Å map of the 70S ribosome from the Lin^s^ strain and a 3.6-Å resolution map of the Lin^r^ 70S ribosome (Fig. S4). The structures were solved initially using a low-resolution 70S model that was built by the use of structure threading protocols in the Rosetta software suite (20), followed by manual loop building. Next, the model was fitted to the electron density maps using guided molecular dynamic simulations as implemented in NAMD2 (21) (Fig. S3). Bond geometry optimization and the final molecular refinement were carried out in real space using the PHENIX software package (22). The accuracy of the resulting atomic models was judged by an all-atom comparison to the recent X-ray crystal structure of the 50S subunit from the *S. aureus* ribosome (PDB 4WCE). This validation experiment showed the accuracy of the cryo-EM data processing, given a root mean square deviation (RMSD) of less than 0.5 Å.

10.1128/mBio.00395-17.1 TEXT S1  Supplemental methods. Download TEXT S1, PDF file, 0.1 MB. Copyright © 2017 Belousoff et al. 2017 Belousoff et al. This content is distributed under the terms of the Creative Commons Attribution 4.0 International license.

10.1128/mBio.00395-17.4 FIG S2  Cryo-EM data and processing. Download FIG S2, PDF file, 0.7 MB. Copyright © 2017 Belousoff et al. 2017 Belousoff et al. This content is distributed under the terms of the Creative Commons Attribution 4.0 International license.

10.1128/mBio.00395-17.5 FIG S3  Data processing flowchart (Lin^s^ 70S). Download FIG S3, PDF file, 1 MB. Copyright © 2017 Belousoff et al. 2017 Belousoff et al. This content is distributed under the terms of the Creative Commons Attribution 4.0 International license.

10.1128/mBio.00395-17.6 FIG S4  Local resolution analysis. Download FIG S4, PDF file, 1.4 MB. Copyright © 2017 Belousoff et al. 2017 Belousoff et al. This content is distributed under the terms of the Creative Commons Attribution 4.0 International license.

While the data have not yet been released, a recent paper by Yusupov, Hashem, and coworkers describes in detail unique features of the Lin^s^ 70S ribosome from *S. aureus* (23). In relation to that structure, our results provide the first comparison of ribosomes from Lin^s^ and Lin^r^; hence, the analysis presented here focuses on the mechanism of linezolid resistance. The ribosomes from Lin^s^ and Lin^r^ differ by a large structural rearrangement in loop 6 of uL3 (Fig. 2b; Fig. S5a). The deletion of Ser145 induced a contraction of the rProtein loop, while most interactions with helix 90 in the 23S rRNA were retained (Fig. 2b).

10.1128/mBio.00395-17.7 FIG S5  Change in known mutational hot spots around the PTC by the uL3 mutation. Download FIG S5, PDF file, 0.7 MB. Copyright © 2017 Belousoff et al. 2017 Belousoff et al. This content is distributed under the terms of the Creative Commons Attribution 4.0 International license.

This dragged the rRNA helix away from the PTC (by ~2 Å), leading to noticeable differences in the architecture of the linezolid binding site (Fig. 2b and d). Comparing the Lin^r^ and Lin^s^ structures revealed a large shift in G^2505^ into the binding site of linezolid (Fig. 2e). This shift was mediated by the change in orientation of G^2581^ in helix 90 due to the change in uL3. This structural shift completely remodeled the linezolid binding cavity (Fig. 2e) and removed a critical hydrogen bond between the ribose backbone (G^2505^) and the 2-oxazolidone moiety of the antibiotic (24), explaining the lower binding efficacy of the drug.

Further structure comparison also revealed subtler differences in rearrangements of the rRNA base orientations in the PTC (Fig. S5b). The rRNA bases undergoing the largest shifts are U^2504^, G^2505^, U^2506^, G^2576^, and U^2584^, all of which either are within or surround the PTC. In other Lin^r^ strains, these bases are common targets for the mutations that directly confer linezolid resistance (Fig. S5c). Our work demonstrates how a clinically relevant mutation in uL3, which is located more than 20 Å away from the drug pocket, propagates alterations through to these rRNA residues surrounding the linezolid binding site. This link between the ΔSer145 mutation in uL3 and mutations in the 23S rRNA leads to the suggestion that, to achieve a linezolid resistance phenotype, staphylococci must acquire a common spatial change in the linezolid binding site. This common architectural deviation in the various linezolid-resistant mutants provides both an experimental framework and a confidence to engage in drug engineering.

### Methods.

More-detailed descriptions of the methods used are available in the supplemental material. All figures were generated with either Pymol (25) or UCSF Chimera (26).

### MIC and IC_50_ assays.

MIC assays were carried out according to the established protocol as described by Andrews (27). Strains were cultured and assayed for antibiotic inhibition in cation-adjusted Mueller-Hinton broth. IC_50_ assays were performed as previously described (28). Briefly, the inhibition effect of linezolid on *S. aureus* ribosomes was tested in a bacterial coupled transcription/translation assay system which measures the expression of the luciferase gene (29). The results were plotted, and IC_50_ values were calculated with the GraFit software package (30).

### Genome sequencing.

Genomic DNA was isolated from overnight cultures using a Qiagen genomic DNA (gDNA) kit. The DNA was then subjected to DNA library preparation following the protocols outlined by Illumina. Short-read DNA sequencing reads (150 bases, paired ends) were collected on an Illumina MiSeq sequencer and assembled in the Geneious software package.

### Ribosome isolation. (i) Lin^s^ 70S ribosomes with tRNA and mRNA (Lin^s^).

*S. aureus* RN4220 (American Type Culture Collection 35556) (31) was grown and disrupted, and the ribosomes were isolated as described previously (24). Ribosome samples were kept in buffer (10 mM HEPES [pH 7.6], 10 mM MgCl_2_, 60 mM NH_4_Cl, 15 mM KCl) and brought to a final concentration of not higher than 1,000 A_260_ ⋅ ml^−1^ and then were flash-frozen for storage at −80°C.

### (ii) Apo-Lin^r^ 70S ribosomes (Lin^r^).

*S. aureus* bacteria (clinical isolate from Alfred Hospital, Melbourne, Australia) were incubated overnight in 5-ml cultures of brain heart infusion broth. After subculture into 4.5 liters of brain heart infusion broth and growth at 37°C until an optical density (600 nm) of 1.5 was reached, cells were harvested by centrifugation and washed in a buffer containing 10 mM Tris-acetate (pH = 8.0), 14 mM magnesium acetate (MgAc_2_), 50 mM KCl, and 1 mM dithiolthreitol (DTT). Cell pellets were flash-frozen with liquid N_2_. Frozen cell pellets were thawed in the presence of a buffer containing 10 mM Tris-acetate (pH = 8.0), 20 mM MgAc_2_, 50 mM KCl, and 1 mM DTT. Lysostaphin (80 µg/ml) and DNase I (80 µg/ml) were added to this solution. This cell slurry was incubated at 37°C for 30 min before emulsification was performed using an Avestin Emulsiflex C3 homogenizer. The cell lysate was clarified by centrifugation (45,000 relative centrifugal force [RCF], 30 min, 4°C), and the crude ribosome particles were collected from the clarified lysate by ultracentrifugation into a sucrose cushion (230,000 RCF, 19 h, 4°C). The crude ribosome pellet was suspended in buffer containing 1.5 M (NH_4_)_2_SO_4_, 20 mM MgAc_2_, 400 mM KCl, and 20 mM Tris-acetate (pH = 8.0). This solution was then subjected to hydrophobic interaction chromatography using 650 M butyl resin. 70S ribosomes were eluted over a linear ammonium sulfate gradient. Fractions containing 70S particles were pooled and pelleted by ultracentrifugation (230,000 RCF, 19 h, 4°C). The resulting clear pellet was resuspended in a buffer containing 20 mM Tris-acetate (pH = 8.0), 15 mM MgAc_2_, 50 mM KCl, and 10% (wt/vol) sucrose. This mixture was then subjected to sucrose gradient centrifugation across a linear gradient spanning 10% to 40% (wt/vol) sucrose. Fractions eluted from the sucrose gradient containing pure 70S ribosomes were pooled and dialyzed against a buffer containing 20 mM HEPES (pH = 7.4), 15 mM MgAc_2_, 50 mM KAc, 10 mM NH_4_Ac, and 0.5 mM DTT. These purified ribosomes were generally at a suitable concentration for immediate application to the transmission electron microscopy (TEM) grids (~300 ng/µl).

### Electron microscopy.

Samples (4 to 5 µl) were applied to a glow-discharged Quantifoil holey carbon grid (Quantifoil GmbH, Großlöbichau, Germany) and were flash-frozen in liquid ethane using an FEI Vitrobot system (FEI, Hillsboro, OR). Data for the Lin^s^ ribosome were collected on a Tecnai Arctica FEI EM operating at 200-kV acceleration voltage and at a nominal level of underfocus (Δ*z* = −1 to −2.7 μm) using a second-generation complementary metal oxide semiconductor (CMOS) back-thinned direct electron detector (Falcon II) 4,096-by-4,096-pixel camera with a calibrated magnification of ×110,000, corresponding to 0.96 Å per pixel at the specimen level. Exposure time was 1.5 s with a dose of 40 e ⋅ Å^−2^. Data for the Lin^r^ ribosome were collected on an FEI Titan Krios EM operating at 300-kV acceleration voltage at defocus values similar to those employed with the Lin^s^ sample and using the same electron detector. The calibrated magnification was ×127,000, corresponding to 1.1 Å per pixel at the specimen level. Exposure time was 1 s with a dose of 45 e ⋅ Å^−2^.

### Data processing.

Movies were integrated with EMAN (32) or UCSF MotionCor2 (33), and CTF estimation was performed with CTFFIND3 (34). Particles were picked from the micrographs using EMAN (32), and particle analysis and final three-dimensional (3D) reconstruction were performed using RELION (19).

### Atomic model refinement.

A model of the *S. aureus* 70S ribosome was created using an *E. coli* 70S model (3J9Z) (35). This was achieved using the RNA threading protocol in the Rosetta software package (20) for generating the rRNA and the Sculptor (22) application in the PHENIX software package combined with loop modeling as implemented in coot (36). The resulting model was then subjected to energy minimization in order to remove any steric clashes. Fitting the model to the cryo-EM electron density map was achieved using the MDFF routine in namd (37). The fitted model was further refined by rounds of manual model building in coot (36) and real-space refinement as implemented in the Phenix software package (22).

10.1128/mBio.00395-17.2 TABLE S1  Model parameters. Download TABLE S1, PDF file, 0.1 MB. Copyright © 2017 Belousoff et al. 2017 Belousoff et al. This content is distributed under the terms of the Creative Commons Attribution 4.0 International license.

### Accession number(s).

The structures were deposited with accession codes PDB 5T7V and EMDB EMD-8369 (Lin^r^) and accession codes PDB 5TCU and EMDB EMD-8402 (Lin^s^).
