# Supplementary material for: Structural Basis for Linezolid Binding Site Rearrangement in the Staphylococcus aureus Ribosome
Source: mBio. 2017 May 9;8(3):e00395-17. doi: 10.1128/mBio.00395-17 (PMC5424203; doi:10.1128/mBio.00395-17)
Supplement: FIG S1 [file mbo002173303sf1.pdf]

a

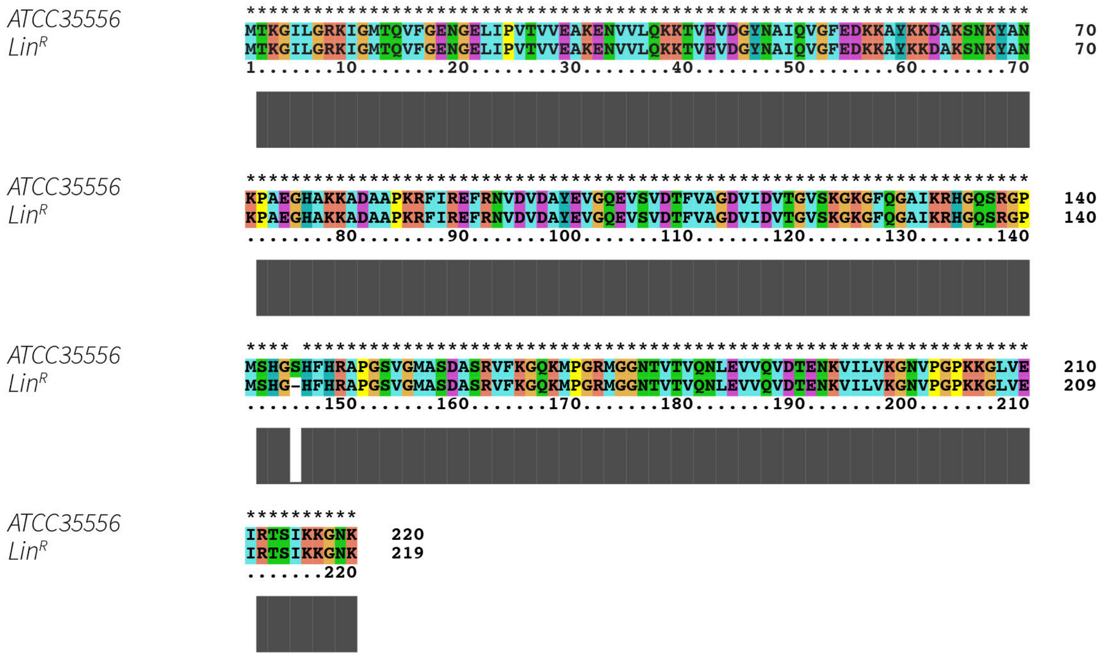

Supplementary Figure S1. The Lin^R mutation in *rpsC*. The protein sequence alignment is based on sequencing of the *rpsC* gene (encoding ribosomal protein uL3) from both *S. aureus* ATCC35556 and from the Lin^R strain.
