# Supplementary material for: Structural Basis for Linezolid Binding Site Rearrangement in the Staphylococcus aureus Ribosome
Source: mBio. 2017 May 9;8(3):e00395-17. doi: 10.1128/mBio.00395-17 (PMC5424203; doi:10.1128/mBio.00395-17)
Supplement: TEXT S1 [file mbo002173303s1.pdf]

SUPPLEMENTARY INFORMATION

MATERIALS AND METHODS

MIC and IC50 assays

MIC assays were carried out according to the established protocol as described by Andrews[1]. Strains were cultured and assayed for antibiotic inhibition in cation adjusted Mueller Hinton Broth.

The EC50 assays were performed as previously described[2]. Briefly, the inhibition effect of linezolid on *S. aureus* ribosomes was tested in a bacterial coupled transcription/translation assay system, in the presence of which measures the expression of the luciferase gene[3]. The luciferase gene was inserted into plasmid with T7 RNA polymerase promoter. The reaction mixture contained: 160 mM Hepes-KOH (pH 7.5), 6.5% PEG 8K, 0.074 mg/mL tyrosine, 1.3 mM ATP, 0.86 mM CTP, GTP and UTP, 208 mM potassium glutamate, 83 mM creatine phosphate, 28 mM NH_4OAc, 0.663 mM cAMP, 1.8 mM DTT, 0.036 mg/mL folinic acid, 0.174 mg/mL *E.coli* tRNA mix, 1 mM amino acid, 0.25 mg/mL creatine kinase, 0.027mg/mL T7 RNA polymerase, ribosome free *E.coli* cell free extract, 300nM of *S. aureus* ribosomes, 0.003 µg/µL luciferase plasmid and linezolid diluted to 233 µg/mL to 149 ng/mL. The reaction mixture was incubated at 37°C for 1hr and terminated by the addition of erythromycin at a final concentration of 8µM. To quantify the reaction's products, Luciferin Assay Reagent (LAR, Promega) at 5:3 (luciferase: reaction mix) volume ratio was added to the mixture and luminescence was measured. The results were plotted and EC_{50} values were calculated with the GraFit software package.[4]

Genome Sequencing

Genomic DNA was isolated from overnight cultures using a Qiagen gDNA kit. The DNA was then subjected to DNA library preparation following the protocols outlined by Illumina. Short reads DNA sequencing reads (150 bases, paired ends) were collected on an Illumina MiSeq sequencer and assembled in the Geneious software package.

Ribosome isolation

apo-Lin^R 70S ribosomes (Lin^R)

Bacterial strains were incubated overnight in 5 mL cultures of brain heart infusion broth. These were subcultured into 4.5 L of brain heart infusion broth and grown at 37 °C until an they reached an optical density (600 nm) of 1.5. The cells were harvested by centrifugation and washed with in a buffer containing 10 mM Tris.Acetate (pH = 8.0), 14 mM Magnesium acetate ($MgAc_2$), 50 mM KCl and 1

mM dithiolthreitol (DTT). Cell pellets were flash frozen with liquid N_2. Frozen cell pellets where thawed in the presence of a buffer containing 10 mM Tris.Acetate (pH = 8.0), 20 mM $MgAc_2$, 50 mM KCl and 1 mM DTT. To this solution was added lysostaphin (80 μg/mL) and DNase I (80 μg/mL). This cell slurry was incubated at 37 °C for 30 minutes before emulsification in an Avestin Emusliflex C3. The cell lysate was clarified by centrifugation (45k rcf, 30 min, 4 °C) and the crude ribosome particles were collected from the clarified lysate by ultracentrifugation into a sucrose cushion (230k rcf, 19 hrs, 4 °C). The crude ribosome pellet was suspended in buffer containing 1.5 M $(NH_4)_2SO_4$, 20 mM $MgAc_2$, 400 mM KCl and 20 mM Tris.acetate (pH = 8.0). This solution was then subjected to hydrophobic interaction chromatography using 650-M Butyl resin. 70S ribosomes were eluted over a linear ammonium sulfate gradient. Fraction containing 70S particles were pooled and pelleted by ultracentrifugation (230k rcf, 19 hrs, 4 °C). The resulting clear pellet was resuspended in a buffer containing 20 mM Tris.Acetate (pH = 8.0), 15 mM $MgAc_2$, 50 mM KCl and 10 % sucrose (w/v). This was then subjected to sucrose gradient centrifugation across a linear gradient spanning 10 – 40 % sucrose (w/v). Fractions eluted from the sucrose gradient containing pure 70S ribosomes were pooled and dialysed against a buffer containing 20 mM HEPES (pH = 7.4), 15 mM $MgAc_2$, 50 mM KAc, 10 mM NH_4Ac and 0.5 mM DTT. These purified ribosomes were generally at a suitable concentration for immediate application to the TEM grids (~ 300 ng/μL).

Lin^S 70S ribosomes with tRNA and mRNA (Lin^S)

S. aureus strain RN4220 (American Type Culture Collection 35556)[5] was grown and disrupted as described previously[6]. Cell extract was layered on a 1.1M sucrose cushion[7], $H_{10}M_{15}N_{100}K_{50}\beta Me_6$ pH 8.0 (10 mM Hepes pH 8.0, 15 mM $MgCl_2$, 100 mM NH_4Cl, 50 mM KCl, 6 mM β-mercaptoethanol) and ultracentrifuged twice, each time for 17hrs at 4 °C at 55k rpm using a Ti-70 rotor. The supernatant was then discarded; the pellet was dissolved in $H_{10}M_{15}N_{150}\beta Me_6$ buffer pH 8.0 at 4 °C. Ribosomal subunits were then separated by zonal ultracentrifugation, using a Ti-15 zonal rotor with a gradient of 8% - 40% sucrose, at low Mg^{2+} concentration (1 mM $MgCl_2$) for 17.5 hrs at 27k rpm. After separation, the Mg^{2+} concentration was adjusted to 10 mM and the ribosomal subunits fractions were concentrated using sequential centrifugations. The samples were kept in $H_{10}M_{10}N_{60}K_{15}$ at pH 7.6, and brought to a final concentration not higher than $1000A_{260}$ $.mL^{-1}$ then were flash-frozen for storage at -80°C.

Electron microscopy

Lin^R ribosomes

5 μL of sample were applied to glow-discharged Quantifoil R2/2 holey carbon grids (Quantifoil GmbH, Großlöbichau, Germany) for preparing frozen-hydrated specimen using a Vitrobot Mark IV

(FEI, Hillsboro, OR) with a 3-s blotting time at 100% humidity. Grids were transferred under liquid nitrogen to a Titan Krios transmission electron microscope (FEI, Hillsboro, OR) operated at 300 kV and set for parallel illumination. 1 s exposures with a calibrated magnification of ~127,000 (corresponding to a pixel size of 1.1 Å on the specimen) were automatically recorded on a Falcon 2 camera (FEI, Hillsboro, OR) in 7-frame movie mode using a dose rate of 45 electrons per second controlled by data acquisition software EPU (FEI, Hillsboro, OR). The corresponding 17 sub-frames were fractionated in 7 frames as follows: Sub-frame 1 was discarded. Sub-frames 2 to 7 were recorded as frames 1 to 6, respectively. Sub-frames 8 to 16 were pooled and integrated as frame 7. Sub-frame 17 was discarded. The defocus was set to a range of 0.6 μm to 3.5 μm in intervals of 0.2 μm. 3353 movies were recorded for the Lin^R ribosomes.

Lin^S 70S ribosomes with tRNA and mRNA

4 μl aliquots of the SA70S ribosome in complex with tRNA and mRNA were incubated for 30 sec on a glow discharged 2-nm carbon-coated holey grids (Quantifoil R2/2). Grids were blotted for 3 sec in 100% humidity at 4 °C and flash frozen in liquid N_2 cooled liquid ethane using an FEI Vitrobot. Data were collected on the Tecnai Arctica FEI EM operating at 200 kV acceleration voltage and at a nominal underfocus of Δz = (−1)- (–2.7) μm using the second-generation back-thinned direct electron detector CMOS (Falcon II) 4,096 × 4,096 camera and automated data collection with EPU software. The Falcon II camera was calibrated at nominal magnification of 110K×. The calibrated magnification on the 14 μm pixel camera was 146 × resulting in 0.96 Å pixel size at the specimen level. The camera was set up to collect 7 frames, plus one total exposure image. Total exposure time was 1.5 s with a dose of 40 e.$Å^{-2}$.

Data processing

Lin^R ribosomes

Movies were integrated in EMAN2 [8] by averaging all 7 frames. RELION 1.4 [9] was used as wrapper for CTF estimation with CTFFIND3 [10] and sorting according to quality of the CTF fit yielded 2846 images for Lin^R ribosome. ~2000 particles were automatically selected for either sample from ~50 Lin^R and images using the swarm tool of the e2boxer.py program of EMAN2. 2D classification of this initial dataset in RELION 1.4 provided class averages that served as templates for particle selection across all retained images using the autopick function in RELION 1.4 yielding ~480,000 Lin^R particles. Particles were extracted with a box size of 400 × 400 pixels and the full dataset classified in RELION 1.4. 10 representative classes were subjected to the e2initialmodel.py program in EMAN2 for generating an initial model. The initial model was low-pass filtered to 60 Å and together with

particles extracted from the best classes provided to 3D classification in RELION 1.4 for disentangling of particles into conformationally homogenous subsets. The combination of 2D and 3D classification yielded ~80,500 Lin^R particles. These final subsets were provided for the final refinement in RELION 1.4 with successive correction movie processing for motion correction. For this purpose, only frames 1 to 6 of the movies was extracted and particle polishing performed without running a window of averaged subframes. Resolution of the final reconstructions was determined using the gold-standard FSC=0.143 criterion See Supplementary Figure S2 and Supplementary Table S1.

Lin^S 70S ribosomes with tRNA and mRNA

Before particle picking, stack alignment was performed, which included 7 frames and total exposure image (total 8 images in the stack). These 8 images in the stack were aligned by the whole image motion correction method,[11] and were evaluated by the look of the power spectra (poor quality images were discarded). Thereafter, an average image of whole stack was used to pick about 220,000 particles semi automatically using EMAN2.1 Boxer,[8] and the contrast transfer function of every image was determined using CTFFIND3[10] in the RELION 1.3[9] workflow. 2D classification was used to remove bad particles (30,000) and particle sorting was done by 3D classification which removed additional bad particle (~64,000). 126,000 particles were used for high-resolution refinement in RELION.

Atomic model refinement

A model of the *S. aureus* 70S ribosome was created using an *E. coli* 70S model (3J9Z)[12]. This was achieved using the RNA threading protocol in the Rosetta software package[13] for generating the rRNA and the Sculptor[14] application in the PHENIX software package combined with loop modelling as implemented in coot[15]. The resulting model was then subjected to energy minimisation in order to remove any steric clashes. Fitting the model to the cryoEM electron density map was achieved using the MDFF routine in namd[16]. The fitted model was further refined by rounds of manual model building in coot[15] and real space refinement as implemented in the Phenix software package[14].

REFERENCES

1 Andrews, J. M. Determination of minimum inhibitory concentrations. *J Antimicrob Chemother* **48 Suppl 1**, 5-16 (2001).

2 Belousoff, M. J., Shapira, T., Bashan, A., Zimmerman, E., Rozenberg, H., Arakawa, K., Kinashi, H. & Yonath, A. Crystal structure of the synergistic antibiotic pair, lankamycin and lankacidin, in complex with the large ribosomal subunit. *Proc Natl Acad Sci U S A* **108**, 2717-2722, doi:10.1073/pnas.1019406108 (2011).

3 Murray, R. W., Melchior, E. P., Hagadorn, J. C. & Marotti, K. R. Staphylococcus aureus cell extract transcription-translation assay: firefly luciferase reporter system for evaluating protein translation inhibitors. *Antimicrobial agents and chemotherapy* **45**, 1900-1904 (2001).

4 Leatherbarrow, R. J. *GraFit Version 7, Erithacus Software Ltd.*, (Horely, U.K., 2010).

5 Novick, R. P. Genetic systems in staphylococci. *Methods Enzymol* **204**, 587-636 (1991).

6 Eyal, Z., Matzov, D., Krupkin, M., Wekselman, I., Paukner, S., Zimmerman, E., Rozenberg, H., Bashan, A. & Yonath, A. Structural insights into species-specific features of the ribosome from the pathogen Staphylococcus aureus. *Proc Natl Acad Sci U S A* **112**, E5805-5814, doi:10.1073/pnas.1517952112 (2015).

7 Selmer, M., Dunham, C. M., Murphy, F. V., Weixlbaumer, A., Petry, S., Kelley, A. C., Weir, J. R. & Ramakrishnan, V. Structure of the 70S ribosome complexed with mRNA and tRNA. *Science* **313**, 1935-1942 (2006).

8 Tang, G., Peng, L., Baldwin, P. R., Mann, D. S., Jiang, W., Rees, I. & Ludtke, S. J. EMAN2: an extensible image processing suite for electron microscopy. *Journal of structural biology* **157**, 38-46, doi:10.1016/j.jsb.2006.05.009 (2007).

9 Scheres, S. H. RELION: implementation of a Bayesian approach to cryo-EM structure determination. *Journal of structural biology* **180**, 519-530, doi:10.1016/j.jsb.2012.09.006 (2012).

10 Mindell, J. A. & Grigorieff, N. Accurate determination of local defocus and specimen tilt in electron microscopy. *Journal of structural biology* **142**, 334-347 (2003).

11 Li, X., Mooney, P., Zheng, S., Booth, C. R., Braunfeld, M. B., Gubbens, S., Agard, D. A. & Cheng, Y. Electron counting and beam-induced motion correction enable near-atomic-resolution single-particle cryo-EM. *Nature methods* **10**, 584-590 (2013).

12 Li, W., Liu, Z., Koripella, R. K., Langlois, R., Sanyal, S. & Frank, J. Activation of GTP hydrolysis in mRNA-tRNA translocation by elongation factor G. *Sci Adv* **1**, doi:10.1126/sciadv.1500169 (2015).

13 Cheng, C. Y., Chou, F. C. & Das, R. Modeling complex RNA tertiary folds with Rosetta. *Methods Enzymol* **553**, 35-64, doi:10.1016/bs.mie.2014.10.051 (2015).

14 Adams, P. D., Afonine, P. V., Bunkoczi, G., Chen, V. B., Davis, I. W., Echols, N., Headd, J. J., Hung, L. W., Kapral, G. J., Grosse-Kunstleve, R. W., McCoy, A. J., Moriarty, N. W., Oeffner, R., Read, R. J., Richardson, D. C., Richardson, J. S., Terwilliger, T. C. & Zwart, P. H. PHENIX: a comprehensive Python-based system for macromolecular structure solution. *Acta Crystallogr D Biol Crystallogr* **66**, 213-221, doi:10.1107/S0907444909052925 (2010).

15 Emsley, P., Lohkamp, B., Scott, W. G. & Cowtan, K. Features and development of Coot. *Acta Crystallogr D Biol Crystallogr* **66**, 486-501, doi:10.1107/S0907444910007493 (2010).

16 Chan, K. Y., Trabuco, L. G., Schreiner, E. & Schulten, K. Cryo-electron microscopy modeling by the molecular dynamics flexible fitting method. *Biopolymers* **97**, 678-686, doi:10.1002/bip.22042 (2012).
