# Supplementary material for: Structural Basis for Linezolid Binding Site Rearrangement in the Staphylococcus aureus Ribosome
Source: mBio. 2017 May 9;8(3):e00395-17. doi: 10.1128/mBio.00395-17 (PMC5424203; doi:10.1128/mBio.00395-17)
Supplement: FIG S2 [file mbo002173303sf2.pdf]

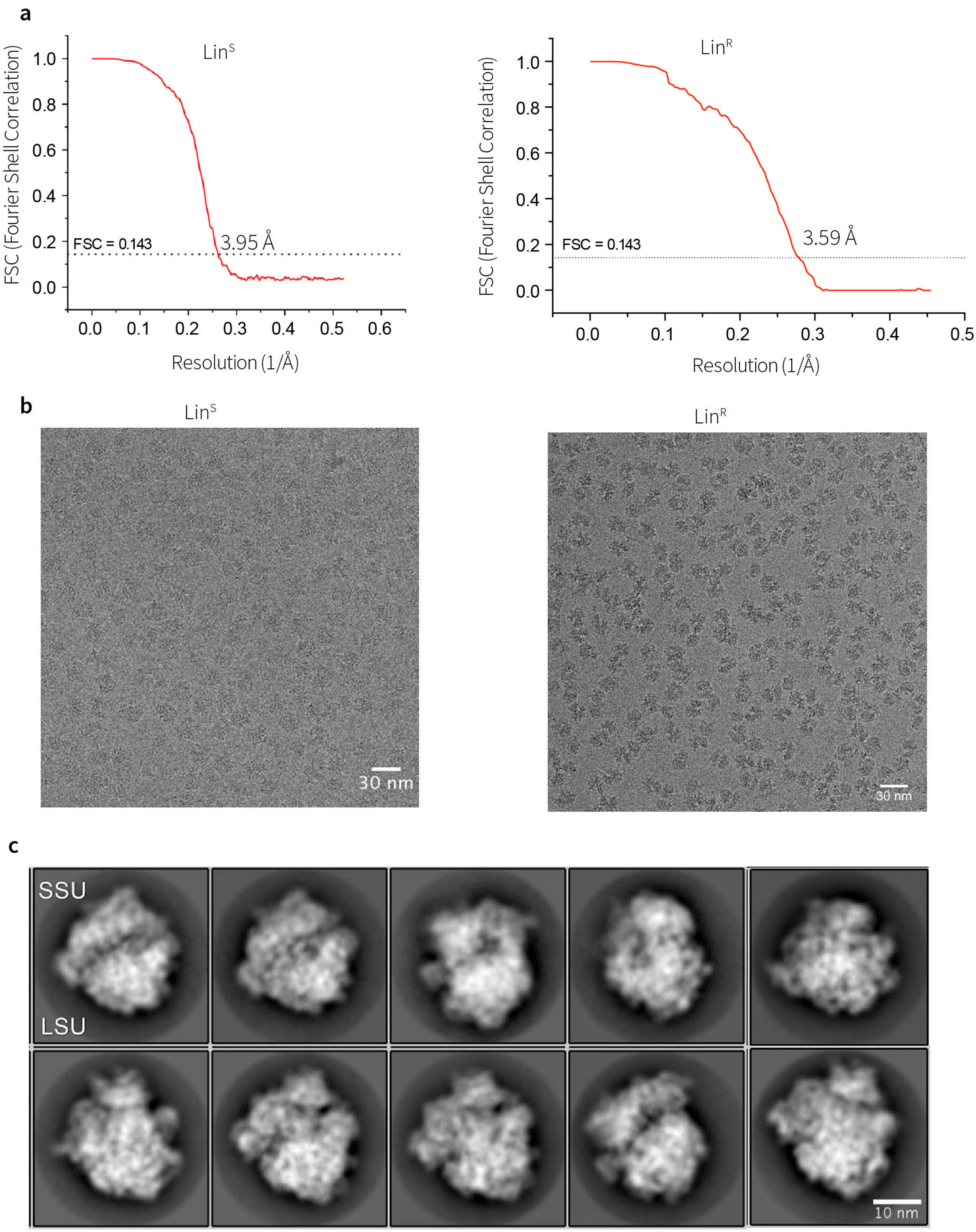

Supplementary Figure S2. Cryo-EM data and processing. a. Fourier Shell Correlation (FSC) plot of the final 3D map indicates a resolution of 3.95 Å for the Lin^S ribosome and 3.59 Å for the Lin^R ribosome (FSC=0.143 criteria). **b.** Representative cryo-EM micrographs showing distribution of 70S ribosomal particles on grid recorded at -1.8 µm defocus (scale bar, 30 nm). **c.** Representative 2D class averages of the Lin^S 70S ribosome showing well defined density for both the LSU and SSU (scale bar, 10 nm).
