# Supplementary material for: Structural Basis for Linezolid Binding Site Rearrangement in the Staphylococcus aureus Ribosome
Source: mBio. 2017 May 9;8(3):e00395-17. doi: 10.1128/mBio.00395-17 (PMC5424203; doi:10.1128/mBio.00395-17)
Supplement: FIG S3 [file mbo002173303sf3.pdf]

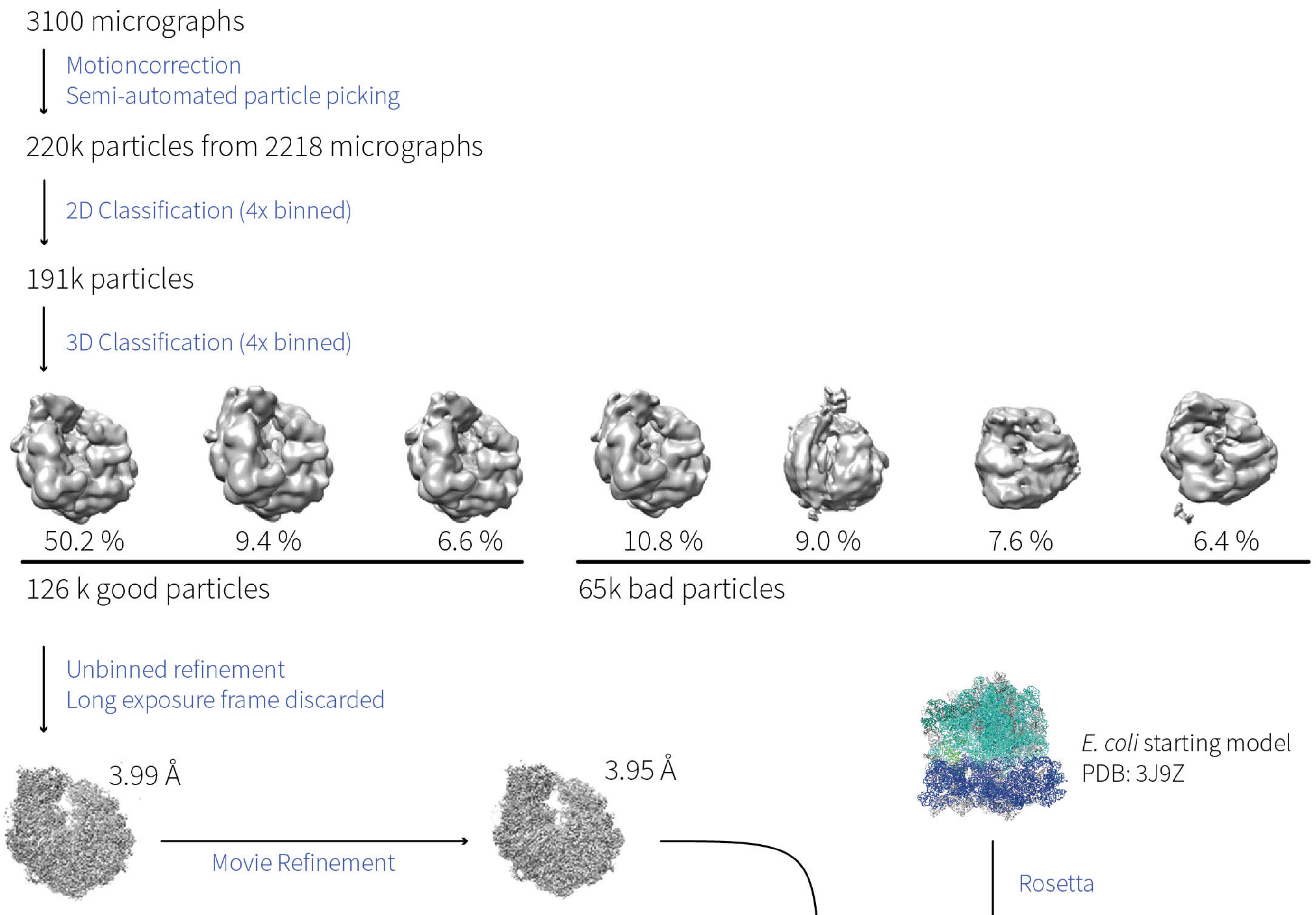

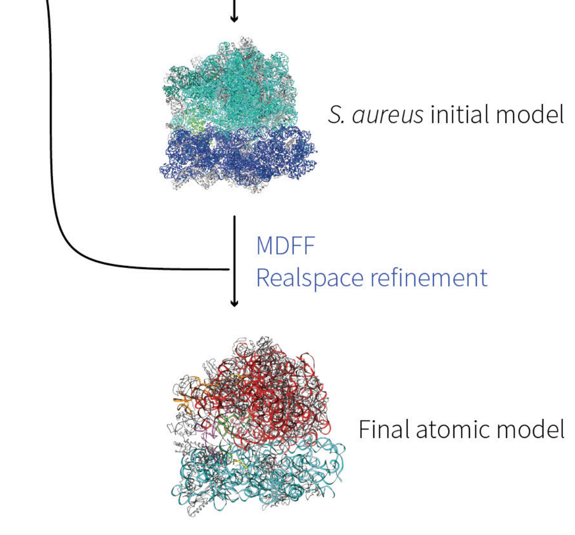

Supplementary Figure S3. Data processing flowchart (Lin^S 70S). A total of 3100 cryo-EM images were collected in a movie-mode containing seven short exposure frames and one long exposure frame. Whole image drift correction was done running motioncorr (Li, X., Mooney, P., Zheng, S., Booth, C. R., Braunfeld, M. B., Gubbens, S., Agard, D. A. & Cheng, Y. Nat Meth 10, 584-590, doi:10.1038/nmeth.2472) on image stacks generated from these eight frames. After screening for good micrographs 2218 micrographs were selected for data processing. Particles were picked semi-automatically using e2boxer.py from EMAN 2.1 (Bell, J. M., Chen, M., Baldwin, P. R. & Ludtke, S. J. Methods 100, 25-34, doi:http://dx.doi.org/10.1016/j.ymeth.2016.02.018 (2016).) and CTF parameters were determined using CTFFIND3 (Mindell, J. A. & Grigorieff, N. Journal of Structural Biology 142, 334-347, doi:http://dx.doi.org/10.1016/S1047-8477(03)00069-8 (2003)). 2D classification, 3D classification and 3D refinement were carried out using RELION 1.3 (Scheres, S. H. W. Journal of Structural Biology 180, 519-530, doi:http://dx.doi.org/10.1016/j.jsb.2012.09.006 (2012)). In total, 220K particles were 4X binned and sorted using 2D classification, discarding 29K particles. 4X binned particles were further sorted using 3D classification using a 60 Å low-pass filtered cryo-EM reconstruction of a T. thermophilus ribosome (Kumar, V., Chen, Y., Ero, R., Ahmed, T., Tan, J., Li, Z., Wong, A. S., Bhushan, S. & Gao, Y. G. Proc Natl Acad Sci U S A 112, 10944-10949, doi:10.1073/pnas.1513216112 (2015)) as reference and classes containing 65K low-resolution ribosomal particles were discarded. 3D refinement was carried out on 126K unbinned particles discarding long exposure frame which resulted in a cryo-EM map at an average 3.99Å resolution. Statistical movie processing (particle polishing) resulted in the final map resolved to 3.95Å. Local resolution of the final masked map was calculated using ResMap (Kucukelbir, A., Sigworth, F. J. & Tagare, H. D. Nat Methods 11, 63-65, doi:10.1038/nmeth.2727 (2014)) with half-reconstructions as input maps, following FSC=0.143 criterion (Rosenthal, P. B. & Henderson, R. Journal of Molecular Biology 333, 721-745, doi:http://dx.doi.org/10.1016/j.jmb.2003.07.013 (2003)). The cryo-EM map was corrected for modulation transfer function (MTF) of Falcon II direct electron detector and sharpened using automatically calculated B-factor (-140 Å2), prior to visualization. Initial model generation was undertaken taking an E. coli 70S ribosome (PDB: 3J9Z Li, W., Liu, Z., Koripella, R. K., Langlois, R., Sanyal, S. & Frank, J. Sci Adv 1, doi:10.1126/sciadv.1500169 (2015)) as the starting model. Threading and minimization protocols as implemented in Rosetta (Cheng, C. Y., Chou, F. C. & Das, R. Methods Enzymol 553, 35-64, doi:10.1016/bs.mie.2014.10.051 (2015).) were used to generate an initial model of the S. aureus ribosome. At this point the model was subjected to molecular dynamics flexible fitting (MDFF) (Phillips, J. C., Braun, R., Wang, W., Gumbart, J., Tajkhorshid, E., Villa, E., Chipot, C., Skeel, R. D., Kale, L. & Schulten, K. Journal of computational chemistry 26, 1781-1802, doi:10.1002/jcc.20289 (2005).) and multiple rounds of manual model building, inspection and realspace refinement (Adams, P. D., Afonine, P. V., Bunkoczi, G., Chen, V. B., Davis, I. W., Echols, N., Headd, J. J., Hung, L. W., Kapral, G. J., Grosse-Kunstleve, R. W., McCoy, A. J., Moriarty, N. W., Oeffner, R., Read, R. J., Richardson, D. C., Richardson, J. S., Terwilliger, T. C. & Zwart, P. H. Acta Crystallogr D Biol Crystallogr 66, 213-221, doi:10.1107/S0907444909052925 (2010).)
