# Supplementary material for: Structural Basis for Linezolid Binding Site Rearrangement in the Staphylococcus aureus Ribosome
Source: mBio. 2017 May 9;8(3):e00395-17. doi: 10.1128/mBio.00395-17 (PMC5424203; doi:10.1128/mBio.00395-17)
Supplement: FIG S4 [file mbo002173303sf4.pdf]

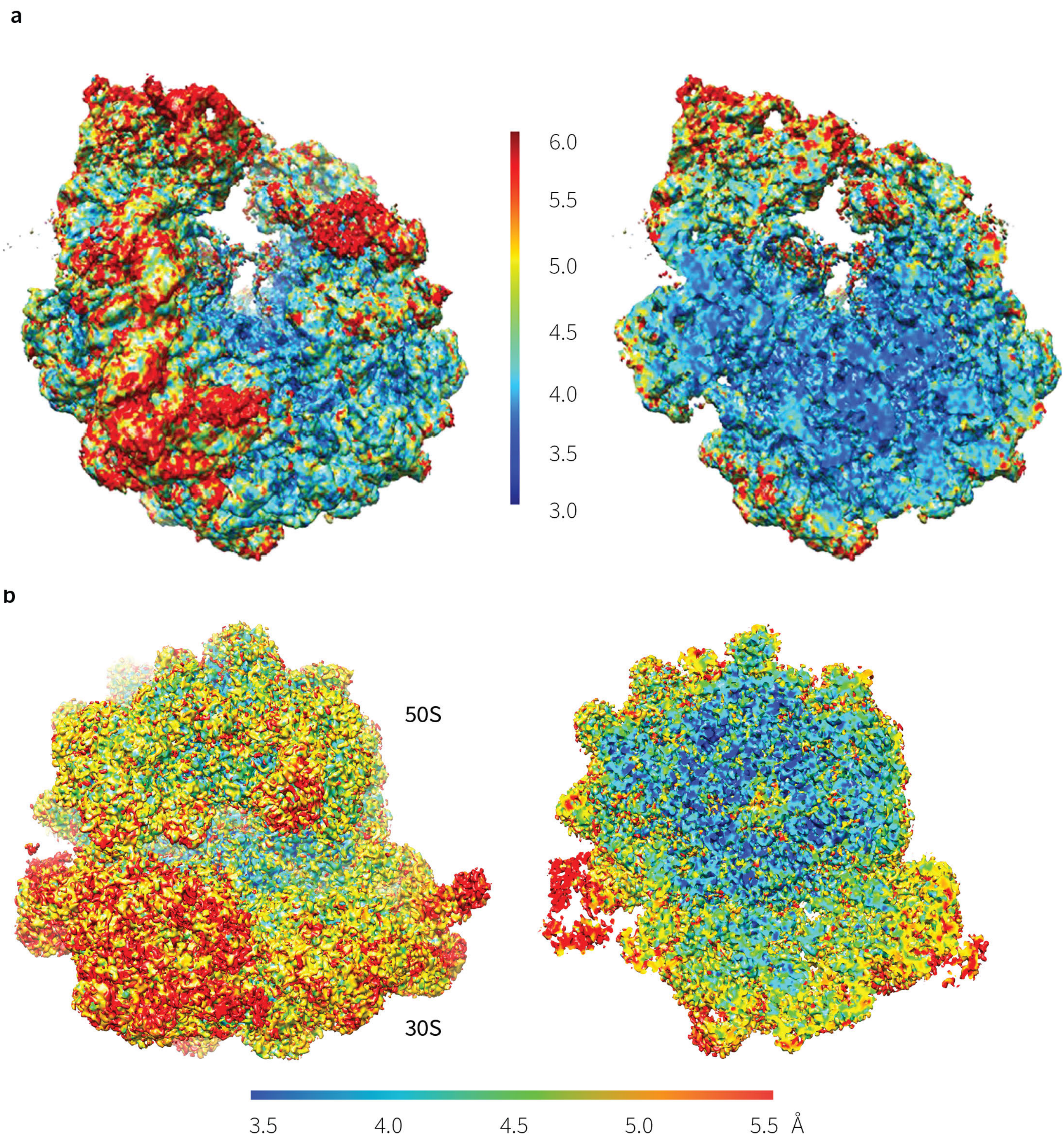

Supplementary Figure S4. Local resolution analysis. a. Cryo-EM density map is shown colored according to the local resolution of the masked map, using ResMap (Kucukelbir, A., Sigworth, F. J. & Tagare, H. D. *Nat Methods* 11, 63-65, doi:10.1038/nmeth.2727 (2014)) for the Lin^S ribosome. **b.** ResMAP analysis of the Lin^R density map, showing local resolution. On the left is the full particle, on the right is a slice directly through the center of the molecule, showing that the local resolution is higher in the core of the particle, compared to the outside.
