# Supplementary material for: Structural Basis for Linezolid Binding Site Rearrangement in the Staphylococcus aureus Ribosome
Source: mBio. 2017 May 9;8(3):e00395-17. doi: 10.1128/mBio.00395-17 (PMC5424203; doi:10.1128/mBio.00395-17)
Supplement: FIG S5 [file mbo002173303sf5.pdf]

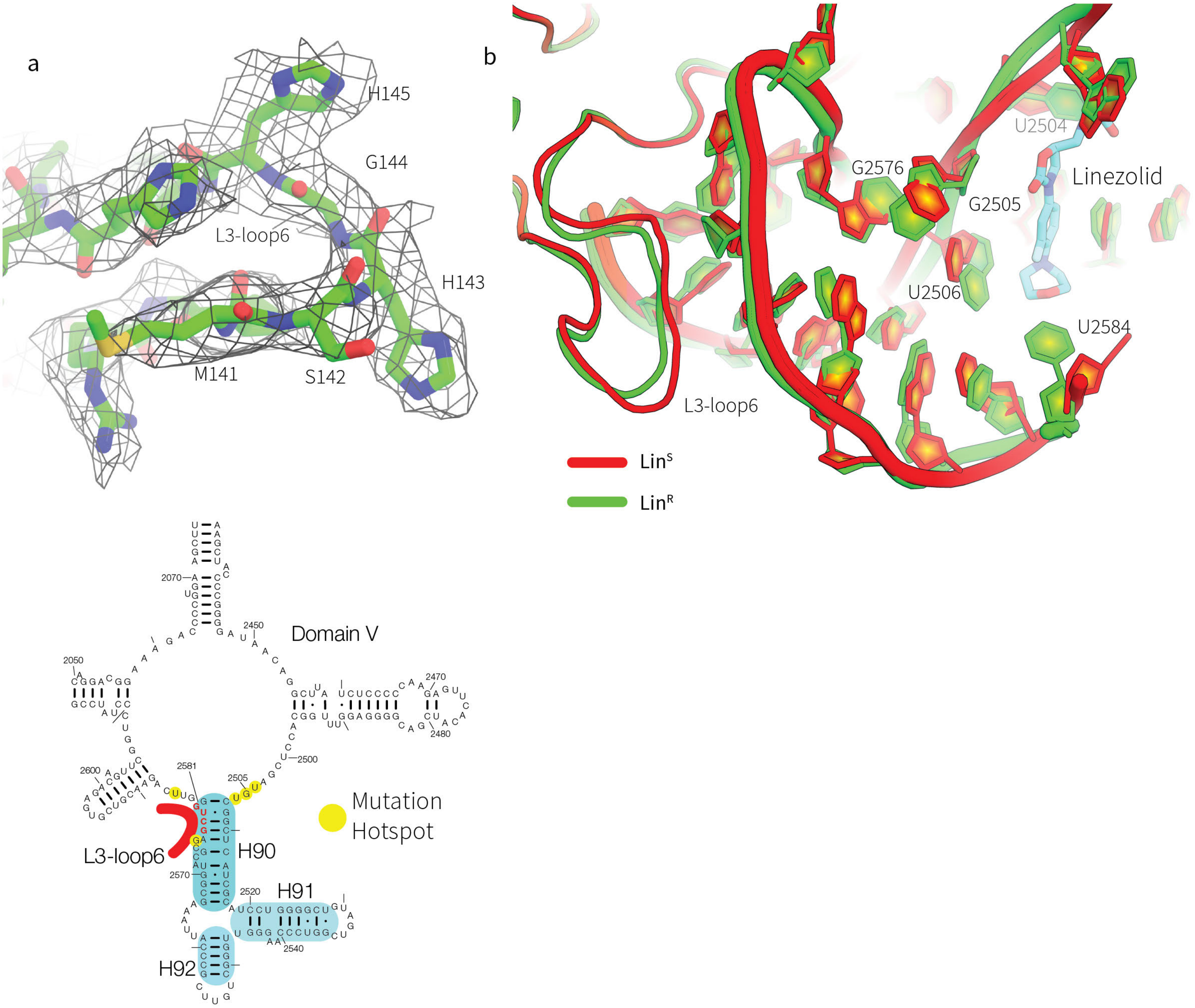

Supplemental Figure S5. ***Change in known mutational hotspots around the PTC by the uL3 mutation.* a.** Electron density map drawn around loop 6 of uL3 in the Lin^{R} ribosome, clearly showing density for the amino acid side chains (Drawn at 3 σ). **b.** Structural alignment of the 70S ribosome from Lin^{S} (red) and the 70S ribosome from Lin^{R} (green), zoomed into the site of both the mutation in uL3 and the linezolid binding site. The notated bases are those that have changed orientation due to the mutation in uL3. **c.** Secondary structure representation of the portion of the 23S rRNA of *S. aureus* that contains both the catalytic site for protein synthesis as well the linezolid binding site. Highlighted in yellow are sites of mutational variability that impart linezolid resistance. In red, is the part of rProtein uL3 that interacts with helix 90 (H90).
