# Supplementary material for: Structural Basis for Linezolid Binding Site Rearrangement in the Staphylococcus aureus Ribosome
Source: mBio. 2017 May 9;8(3):e00395-17. doi: 10.1128/mBio.00395-17 (PMC5424203; doi:10.1128/mBio.00395-17)
Supplement: TABLE S1 [file mbo002173303st1.pdf]

Supplementary Table S1. Model Parameters

**Data Collection**	*Lin*^{R}	*Lin*^{S}
Particles	80,500	126,000
Pixel size (Å)	1.1	0.96
Defocus range (µm)	0.6-3.5	1.0-2.7
Voltage (kV)	300	200
Electron dose (e/Å^2)	45	40
**Refinement**		
CC_{map_model}	0.74	0.73
**Model Quality**		
RMSD		
Bond length (Å) / Bond angles (°)	0.008 / 0.98	0.009/1.1
Ramachandran		
Most favored (%)	88.1	82.9
Outliers (%)	0.12	0.30
Favoured (%)	11.8	16.8
Rotamer outliers	0.19	0.83
C-Beta deviations	0	0
Clashscore	3.63	5.76
